# Influence of sexual dimorphism on satellite cell regulation and inflammatory response during skeletal muscle regeneration

**DOI:** 10.14814/phy2.15798

**Published:** 2023-10-05

**Authors:** Charline Jomard, Julien Gondin

**Affiliations:** ^1^ Institut NeuroMyoGène (INMG), Physiopathologie et Génétique du Neurone et du Muscle (PGNM), Université Claude Bernard Lyon Lyon France

**Keywords:** estrogens, inflammation, satellite cells, skeletal muscle

## Abstract

After injury, skeletal muscle regenerates thanks to the key role of satellite cells (SC). The regeneration process is supported and coordinated by other cell types among which immune cells. Among the mechanisms involved in skeletal muscle regeneration, a sexual dimorphism, involving sex hormones and more particularly estrogens, has been suggested. However, the role of sexual dimorphism on skeletal muscle regeneration is not fully understood, likely to the use of various experimental settings in both animals and human. This review aims at addressing how sex and estrogens regulate both the SC and the inflammatory response during skeletal muscle regeneration by considering the different experimental designs used in both animal models (i.e., ovarian hormone deficiency, estrogen replacement or supplementation, treatments with estrogen receptors agonists/antagonists and models knockout for estrogen receptors) and human (hormone therapy replacement, pre vs. postmenopausal, menstrual cycle variation…).

## 1 INTRODUCTION

Muscle damage may occur after unaccustomed muscle contractions, especially when exercise involves repeated eccentric contractions at high intensities and/or over a large range of motion or isometric contractions induced by neuromuscular electrical stimulation at long muscle length (Clarkson & Hubal, 2002; Fouré & Gondin, 2021). This condition is usually defined as exercise‐induced muscle damage (EIMD). In human, EIMD is usually assessed from indirect markers such as delayed onset muscle soreness (DOMS), increased levels of plasmatic enzymes (e.g., creatine kinase [CK]), a decreased maximal joint range of motion and a long‐lasting reduction of force production, the latter being considered as the best indirect marker of muscle damage (Paulsen et al., 2012; Warren et al., 1999). Histological analysis after EIMD may reveal disruptions of the myofibrillar structure (z‐line streaming, increase of desmin‐negative myofibers), necrosis (at least in animal models of EIMD) and cellular infiltration (Clarkson & Hubal, 2002; Crameri et al., 2007).

Upon muscle damage, satellite cells (SC, skeletal muscle stem cells), which are located under the basal lamina of myofibers, are indispensable for muscle regeneration (Lepper et al., 2011; Murphy et al., 2011; Sambasivan et al., 2011). Indeed, SCs get activated, proliferate, commit to terminal myogenic differentiation and eventually fuse to rebuild new functional myofibers (Yin et al., 2013). Proper muscle regeneration relies on the dynamic interplay between SCs and their microenvironment including immune cells (macrophages), endothelial cells and fibro‐adipogenic progenitors (FAPs)/fibroblasts (Bentzinger et al., 2013). Inflammatory response plays a key role in this process (Arnold et al., 2007). Indeed, the recruited circulating monocytes differentiate into pro‐inflammatory macrophages, which enhance SC proliferation before switching their phenotypes to anti‐inflammatory macrophages, to promote their differentiation and fusion to regenerate injured myofibers (Arnold et al., 2007). Endothelial cells and FAPs also participate to the regeneration process by promoting SC proliferation and extracellular matrix remodeling after damage, respectively (Christov et al., 2007; Lemos et al., 2015). Readers are invited to refer to recent reviews on the cellular interactions and the molecular effectors involved in muscle regeneration (Bernard et al., 2022; Chazaud, 2020; Panci & Chazaud, 2021).

Among the mechanisms involved in skeletal muscle damage and regeneration, a sexual dimorphism has been suggested (Amelink et al., 1990; Amelink & Bär, 1986; Deasy et al., 2007; Enns & Tiidus, 2008; Hubal & Clarkson, 2009; Stupka et al., 2000; Tiidus & Enns, 2009; Velders et al., 2012). The pioneering study of Amelink and colleagues showed that males displayed a higher resting CK activity as compared with females (Amelink et al., 1990). In addition, while CK activity remained unchanged in females after EIMD (Amelink & Bär, 1986), this parameter was significantly elevated in both males and ovariectomized females, illustrating that ovarian hormones might have a protective effect on muscle damage. It has been further suggested that hormonal regulation, and more particularly a greater estrogen production, may have a positive impact on the regulation of SC fate and inflammatory response (Enns & Tiidus, 2008; Liao et al., 2019).

Briefly, estrogens (i.e., estrone [E1], 17β‐estradiol [E2] and estriol [E3]), a group of sex hormones, are involved in the development and regulation of the female reproductive system. Their main source of production and secretion is the granulose cells of the ovaries while other tissues (adrenal glands, liver, fat, skeletal muscle) contribute to a lesser extent to circulating estrogens. E2 is the most biologically active form of estrogen and therefore the most widely investigated. Estrogens exert their function through three different estrogen receptors (ER) (Collins et al., 2019; Enns et al., 2008; Seko et al., 2020; Velders et al., 2012): ER‐α, ER‐β, and G protein‐coupled receptor (GPER). The biological effects of estrogens are mediated by genomic mechanisms through their binding to cytosolic ER. This leads to the translocation to the nucleus and the binding of the estrogen‐estrogen receptor complex to estrogen response element in the promotor and enhancer regions of the target genes, ultimately resulting in the transcription of estrogen responsive genes. Estrogens can also act through non‐genomic mechanisms involving intracellular signaling pathways after GPER activation (Arnal et al., 2017).

So far, the role of sexual dimorphism on skeletal muscle regeneration has been investigated using various experimental settings, including for instance ovarian hormone deficiency, E2 replacement (i.e., when E2 is given to ovariectomized animals) or supplementation (i.e., when E2 is given to healthy animals), mouse models knockout for ER or observational human studies. Although recent reviews focused on the impact of E2 on skeletal muscle mass, regeneration and mitochondrial function (McMillin et al., 2022; Pellegrino et al., 2022), no clear distinction was made between the above‐mentioned methodologies, which may explain some controversies in this research field.

This review aims at addressing how sex and estrogens regulate both the SC and the inflammatory response during skeletal muscle regeneration by considering the different experimental designs used in both animal models (i.e., ovarian hormone deficiency, E2 replacement or supplementation, treatments with estrogen receptors agonists/antagonists and models knockout for ER) and human (hormone therapy replacement, pre vs postmenopausal, menstrual cycle variation…). Finally, we provided a brief critical analysis of these different experimental conditions and proposed perspectives on how future studies could most effectively evaluate the impact of sex/estrogen on skeletal muscle regeneration.

## 2 SATELLITE CELLS

### 2.1 Animal studies

**FIGURE 1:**
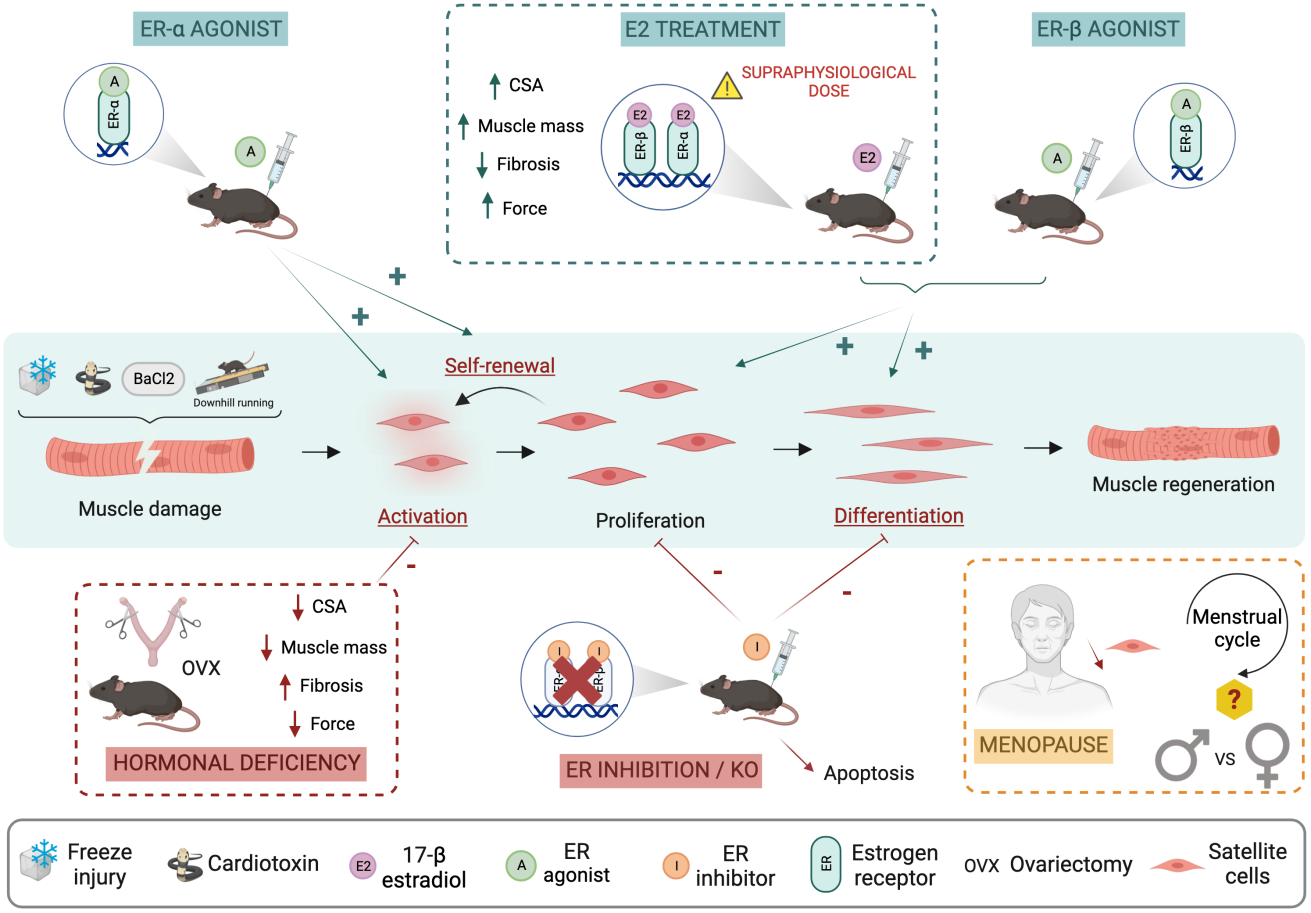
Effects of sex and estrogens on satellite cell fate and skeletal muscle regeneration after injury. After injury, satellite cells (SC) get activated, proliferate, commit to terminal myogenic differentiation and eventually fuse to rebuild new functional myofibers. Ovarian hormone deficiency and estrogen receptors (ER) inhibition/KO affect the different steps of myogenesis (For the sake of clarity, OVX‐induced impairment in SC self‐renewal and differentiation is indicated in red, underlined font). This ultimately leads to impaired muscle regeneration with decreased myofiber cross‐sectional area (CSA) and muscle mass, increased fibrosis and impaired force production. On the contrary, 17β‐estradiol, though often delivered at a supraphysiological dose, and ER‐agonists promote SC activation, proliferation and differentiation, leading to increased myofiber CSA, muscle mass, force and reduced fibrosis. Human studies are rare with some evidence illustrating a decline in SC content from peri to postmenopausal. The influence of both sex and menstrual cycle on SC fate and skeletal muscle regeneration remains to be carefully investigated in human.

The regulation of SC fate after muscle damage has been assessed in different contexts such as ovarian hormone deprivation (i.e., ovariectomy [OVX]) combined or not with E2 replacement and in estrogen receptor knockout‐mice (Figure 1). On the contrary, the impact of sex (i.e., direct comparison between males and females) on SC fate during muscle regeneration has been scarcely investigated (Seko et al., 2020).

#### 2.1.1 Effects of ovarian‐hormone deficiency

Ovariectomy (OVX) is a surgical procedure where ovaries are removed, which leads to an ovarian hormonal deficiency (i.e., including E2) (Souza et al., 2019). A consensus is emerging illustrating that OVX‐induced hormonal deficiency affects the number of SCs at steady‐state and after damage (Collins et al., 2019; Enns & Tiidus, 2008; Kitajima & Ono, 2016; Seko et al., 2020; Velders et al., 2012). The total number of SCs assessed by flow cytometry (i.e., CD31^−^,CD45^−^,VCAM^+^,α7‐integrin^+^) or by immunostaining (i.e., Pax7^+^ cells) was reduced in several uninjured muscles, such as *tibialis anterior*, *extensor digitorum longus*, *gastrocnemius* and *diaphragm*, after ovarian hormone deficiency lasting from 14 days to 7 months (Collins et al., 2019; Kitajima & Ono, 2016; Larson et al., 2022). On the contrary, the SC content of the slower *soleus* muscle was unaffected, suggesting that OVX could specifically affect SC number of the fast muscles (Collins et al., 2019). The number of Pax7^+^ cells was also not affected by OVX when assessed from freshly isolated *extensor digitorum longus* myofibers (Kitajima & Ono, 2016). However, this result should be interpreted with caution as the number of isolated myofibers stained for Pax7 was not reported in this study. Interestingly, SCs obtained from isolated myofibers and cultured for 3 days showed reduced self‐renewal and differentiating capacities after OVX (Kitajima & Ono, 2016). In addition, SCs from OVX mice showed a reduced capacity to transition from G0/G1 to S and G2/M phases as compared with SCs extracted from intact female mice (Larson et al., 2022). This was associated with an upregulated gene expression of *ccna2* and *p16* ^INK4a^, known as key cell cycle regulators. OVX also resulted in a lower engraftment of SCs and a reduction in myogenic cell differentiation and fusion when intact SCs were transplanted in OVX mice as compared to controls (Collins et al., 2019). Interestingly, SCs extracted from OVX donors and transplanted into control mice showed similar engraftment than SCs obtained from control, illustrating the influence of circulating E2 on SC regulation (Collins et al., 2019). Finally, OVX mice showed decreased muscle weight, reduced myofiber cross‐sectional area (CSA) and increased content of collagen type I (an index of fibrosis) as compared with intact females after cardiotoxin (CTX)‐induced muscle damage (Kitajima & Ono, 2016). Overall, OVX‐induced alterations of both SC number and fate led to impaired muscle regeneration.

#### 2.1.2 Effects of E2 replacement and supplementation

E2 replacement rescues the total number of SCs (Collins et al., 2019) and the CSA of fast‐type myofibers (Kitajima & Ono, 2016), which both decreased after 7 months post‐OVX. In vitro studies showed that isolated SCs from intact female mice have greater proliferation, differentiation and fusion index when cultured in the presence of E2 as compared with normal medium (Larson et al., 2022). In the same way, male bovine SCs treated with E2 also showed a higher number of proliferating SCs (Kamanga‐Sollo et al., 2008, 2004). The effects of E2 on SC regulation in vitro were related to IGF‐1‐dependent (Chakravarthy et al., 2000; Kamanga‐Sollo et al., 2004) and ‐independent mechanisms (Kamanga‐Sollo et al., 2008; Pampusch et al., 2003) inasmuch as the use of a competitive inhibitor of IGF‐I binding to type‐I IGF receptor partially reduced E2‐stimulated proliferation in bovine SC cultures.

The role of E2 replacement has been further highlighted in response to muscle damage. SC content of both fast and slow‐type muscles (i.e., *white vastus, white gastrocnemius* and *soleus* muscles) was higher in both males and OVX females rats treated with E2 as compared with non‐treated animals 3 days after downhill running (Enns & Tiidus, 2008; Mangan et al., 2014, 2015; Tiidus et al., 2005). In the same way, SC number and muscle mass were higher and strength recovery was faster in OVX females treated with E2 as compared with untreated females following repeated muscle damage induced by BaCl_2_ injections (Collins et al., 2019; Larson et al., 2020). Also, early administration of E2 (i.e., within 2 weeks post‐OVX) increases the number of SCs 3 days after treadmill running as compared with non‐treated females while late administration (i.e., starting 11 weeks post‐OVX) prevents this beneficial effect (Mangan et al., 2015). In addition, inhibition of the phosphatidylinositol‐3 kinase (PI3K)/Akt pathway hinders E2‐induced SC proliferation both in vitro (Kamanga‐Sollo et al., 2008) and in vivo (Mangan et al., 2014), illustrating that the influence of E2 on SC regulation are mediated, at least in part, by this intracellular signaling system (Chakravarthy et al., 2000).

In summary, E2 supplementation/replacement in cell culture or in OVX animals illustrated the positive role of this hormone on SC regulation during myogenesis and skeletal muscle regeneration.

#### 2.1.3 Modulation of ER

The pioneering study of Enns and colleagues (Enns et al., 2008) demonstrated that downhill running induced higher content of total (Pax7^+^), proliferating (BrdU^+^) and differentiating (MyoD^+^) SC in OVX rats treated with E2 as compared with untreated females. This beneficial effect of E2 was totally prevented by the administration of a specific ER inhibitor (i.e., ICI 182780). Similar inhibitory effects were also demonstrated in vitro on bovine SC proliferation (Kamanga‐Sollo et al., 2008). In addition, protein levels of proliferating cell nuclear antigen and *MyoD* mRNA expression, used as markers of SC activation and differentiation, were higher in controls and in OVX animals treated with either E2 or ER‐β agonist as compared with untreated OVX or those treated with ER‐α agonist (Velders et al., 2012) 3 days after muscle damage induced by notexin (NTX) injection. On that basis, it has been suggested that SC fate could be mainly mediated by ER‐β. However, this result should be considered with caution since ER‐β agonist concentration was ten‐fold higher than the concentration of ER‐α agonist. Interestingly, ER‐β whole‐body knockout (KO) females showed lower mRNA expression of *Pax7, Ki67* and *MyoD* (i.e., markers of SC status) as well as embryonic myosin heavy chain (i.e., a marker of muscle regeneration) compared with either controls or ER‐α whole‐body KO females at 3 days post‐damage (Velders et al., 2012). This illustrates that muscle regeneration could be preferentially mediated by ER‐β. Indeed, E2 or propyl pyrazole triol (i.e., a specific ER‐α agonist) treatment in females resulted in a higher percentage of Pax7^+^ or MyoD^+^ myofibers 3 days after downhill running (Thomas et al., 2010).

Over the last few years, our knowledge on the role of ER on muscle regeneration has been greatly improved thanks to the development of mouse models specifically ablated for ER‐α or ER‐β in SCs. Although not directly quantified, *ERα* protein expression in whole skeletal muscle does not seem to differ between male and female mice (Morfoisse et al., 2018). *ERα* gene expression in whole muscle was the most abundant followed by *Gper* and *ERβ* (Baltgalvis et al., 2010; Couse et al., 1997) in intact female mice while mRNA expression and protein levels of ER are also dependent on myofiber‐type composition (Baltgalvis et al., 2010; Gustafsson et al., 1984; Lemoine et al., 2002; Saartok, 1984), training status (Lemoine et al., 2002) and E2 circulating levels (Baltgalvis et al., 2010).

Nevertheless, the gene encoding for the ER‐α (*Esr1*) is seven times more expressed in SCs than the ones encoding for the ER‐β and GPER (*Esr2* and *Gper1*) (Collins et al., 2019; Larson et al., 2022). Thus, ablation of ER‐α in SC leads to a 40%–60% reduction in SC number for females which was not rescued by E2 replacement, indicating that ER‐α is involved in the maintenance of SC pool (Collins et al., 2019; Larson et al., 2022). Engraftment of SC was also lower in the absence of ER‐α in donor SCs (Collins et al., 2019). Moreover, loss of ER‐α in females leads to SC apoptosis as illustrated by the increased number of Pax7^+^TUNEL^+^ cells, the upregulation of genes involved in cell death (e.g., *H19*, *Fndc1*, mir378c) together with the downregulation of genes promoting cell survival (e.g., *Dhcr24*, *Snord65*, *mir485*) (Collins et al., 2019). Finally, and despite its low mRNA expression (Collins et al., 2019), deletion of *ERβ* in SC resulted in impaired muscle regeneration in females, but not in males, after BaCl_2_ injury (Seko et al., 2020). This was illustrated by reduced myofiber CSA and increased type I collagen content (Seko et al., 2020). These alterations were not exacerbated by OVX, indicating that E2 regulates the SC function through ER‐β during muscle regeneration. ER‐β also regulates SC proliferation and apoptosis while differentiation and fusion of SC seem to be independent of *ERβ* expression, even though it is not totally clear how the latter steps of myogenesis were experimentally characterized (Seko et al., 2020).

To conclude, E2 do exert a positive effect on muscle regeneration, particularly by modulating SC fate in females (Collins et al., 2019; Enns & Tiidus, 2008; Kitajima & Ono, 2016; Seko et al., 2020; Tiidus et al., 2005; Velders et al., 2012). Both ER‐α and ER‐β are implicated in SC function even though their respective contribution to SC fate remains to be determined. In addition, although the influence of E2 on SC fate is now well‐documented in response to toxic injury, their role in EIMD remains unclear.

### 2.2 Human studies

Contrary to animals, the effects of sexual dimorphism on SC number and fate in human are less documented (Figure 1) (Bonavaud et al., 1997; Collins et al., 2019; Fortino et al., 2022; Horwath et al., 2021; Kadi et al., 2004; Roth et al., 2000).

#### 2.2.1 Effect of sex

No sex difference was reported for ER mRNA expression, *ESR1* gene expression being 180‐fold higher than *ESR2* mRNA levels in both men and women (Lemoine et al., 2003), as well as for the proportion of ERα and ERβ positive nuclei (Wiik et al., 2009).

Conflicting findings have been reported on the number of Pax7^+^ cells in men and women at steady state. While several studies showed no difference in SC number between men and women in the *vastus lateralis* and *tibialis anterior* muscles (Binet et al., 2023; Fortino et al., 2022; Kadi et al., 2004; Roth et al., 2000), other investigations reported that men had more Pax7^+^ SCs in type II myofibers as compared with women (Abou Sawan et al., 2021; Horwath et al., 2021). In vitro investigations showed similar proliferation and fusion rates between sexes in four different muscles (i.e., *tensor fasciae*, *vastus lateralis and medialis* and *rectus femoris)* (Bonavaud et al., 1997).

To our knowledge, only one study investigated the effects of EIMD on SC regulation between men and women (Fortino et al., 2022). Men had more Pax7^+^ cells than women 2 days after repeated maximal voluntary eccentric contractions while the number of Pax7^+^/MyoD^+^ cells was not different between sexes. However, these findings should be interpreted with caution as the extent of muscle damage was not accurately compared between men and women (e.g., using force recovery as the best indirect marker of muscle damage (Paulsen et al., 2012)). Further analysis is necessary to carefully determine the impact of sex on SC regulation after EIMD in human.

#### 2.2.2 Effect of E2 level fluctuations in pre and postmenopausal women

The expression of ER varies during the menstrual cycle (Ekenros et al., 2017; Haines et al., 2018). Indeed, both mRNA and protein levels of ERα in skeletal muscle were highest in the follicular phase when circulating estradiol levels were low (Ekenros et al., 2017; Haines et al., 2018). The authors however failed to detect *ESR2* mRNA expression and did not analyze the related protein expression so that it remains to determine whether and to what extent menstrual cycle affects ERβ protein expression (Ekenros et al., 2017). Despite similar E2 muscle concentrations, a greater skeletal muscle *CCND1* mRNA expression was observed in the mid‐follicular phase as compared with the mid‐luteal phase after repeated lengthening contractions. On the contrary, *MYOD1* mRNA expression similarly increased during the two phases after this exercise (Haines et al., 2018). Overall, it is unclear how and to what extent menstrual cycle affects skeletal muscle regeneration.

During the menopausal transition, ovarian hormone levels decrease leading to a reduction in muscle mass and strength production (Collins et al., 2019). Of interest, SC number declined in five women from peri to postmenopausal and was correlated with the reduction of E2 blood concentration (Collins et al., 2019). However, this result needs to be confirmed on a larger sample size (Collins et al., 2019) while it remains to determine how the reduced SC content associated with menopause affects skeletal muscle regeneration.

Overall, the impact of sexual dimorphism on SC regulation during skeletal muscle regeneration requires additional human studies, with a careful control of menstrual cycle.

## 3 INFLAMMATORY RESPONSE

### 3.1 Animal studies

**FIGURE 2:**
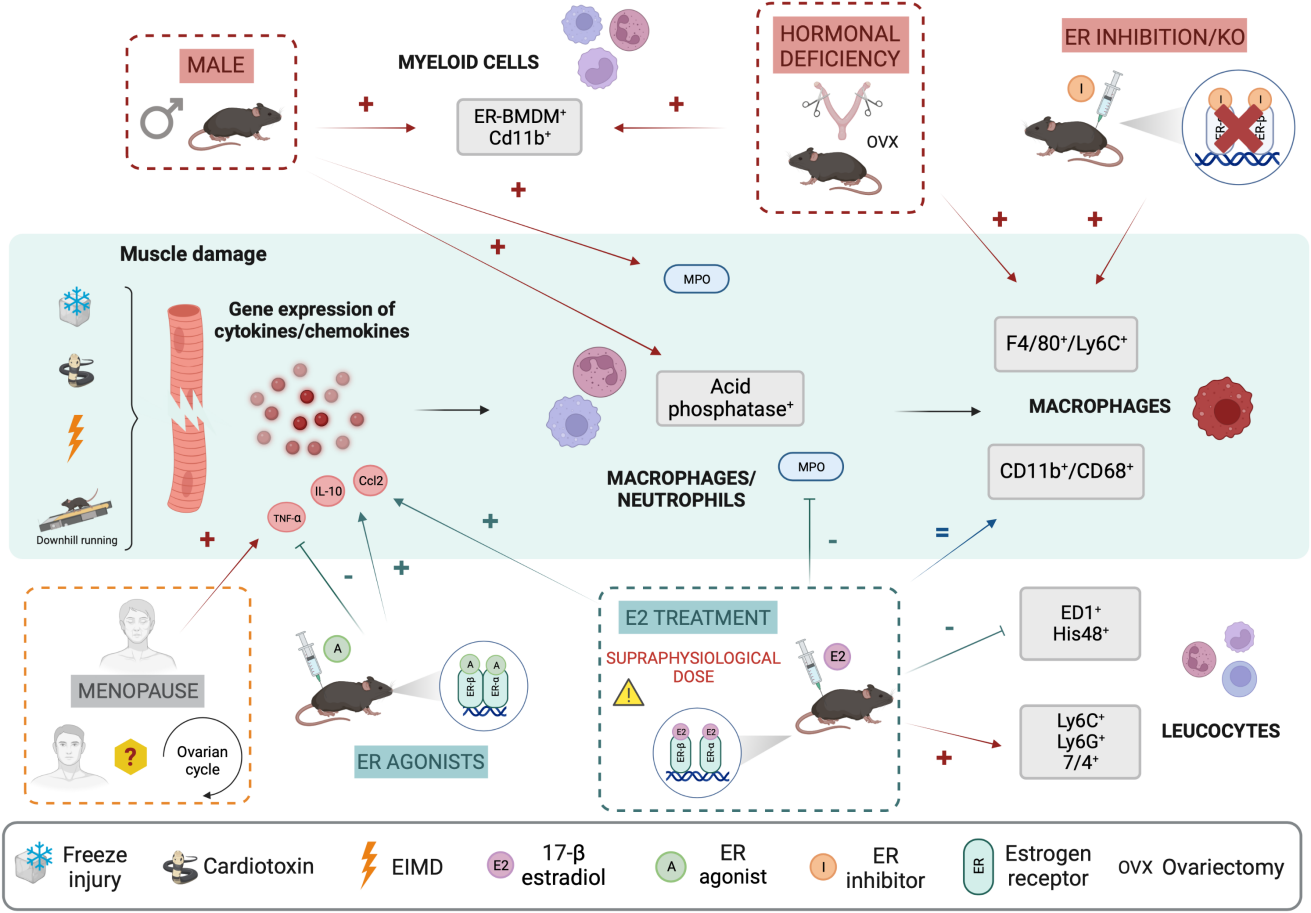
Effects of sex and estrogens on the inflammatory response during skeletal muscle regeneration. After injury, the production of cytokines/chemokines by the damaged myofibres induces the recruitment of immune cells such as neutrophils and macrophages in order to promote skeletal muscle regeneration. In both animal and human studies, the role of sex/ovarian hormones on the inflammatory response after injury is still controversial mainly due to the use of different markers to characterize immune cell infiltration and different models of muscle damage. MPO, myeloperoxidase activity.

Animal experiments investigated the influence of either sex or E2 on the regulation of both immune cell infiltration and their inflammatory status after muscle damage (Figure 2).

#### 3.1.1 Effect of sex

Male rats showed an increase in myeloperoxidase activity (MPO), an enzyme secreted by neutrophils and macrophages, in both *soleus* and *plantaris* muscles while MPO activity remains unchanged in females 1 day after downhill running exercise (Tiidus & Bombardier, 1999). In the same way, the number of myofibers invaded by acid phosphatase^+^ cells (i.e., which stain macrophages and lysosomes) was higher and the infiltration of interstitial ER‐BMDM1^+^ cell (i.e., a marker of myeloid cells) occurs earlier in males as compared with females 1 day after electrically‐evoked lengthening contractions (St Pierre Schneider et al., 1999). On the contrary, no sex difference was observed for the number of leukocytes (Schneider et al., 2012) and CD11b^+^ cells (i.e., a pan marker of myeloid cells) invading damaged myofibers or interstitial spaces after electrically‐evoked lengthening contractions (St Pierre Schneider et al., 1999). Similarly, the number of neutrophils (i.e., CD11b^+^/Gr‐1^+^ cells) and monocytes/macrophages (i.e., CD11b^+^/Gr‐1^−^ cells) as well as the protein levels of monocyte chemoattractant protein 1 and 5 were not different between sex at 3 to 7 days post‐CTX injury (McHale et al., 2012). This latter study also reported that the removal of necrotic myofiber was faster and the extent of fat deposition was greater in females than in males (McHale et al., 2012).

#### 3.1.2 Effect of ovarian‐hormone deficiency and E2 replacement/supplementation

The influence of OVX‐induced ovarian‐hormone deficiency on inflammatory response post muscle injury has been scarcely investigated. No difference was observed for leukocyte infiltration (i.e., assessed by 7/4^+^ cells) between OVX and intact females 1 day after electrically‐evoked lengthening contractions (Schneider et al., 2012) while the proportion of pro‐inflammatory macrophages (i.e., F4/80^+^Ly6C^+^ cells) and the gene expression of *Il1*, *Ccl2* and *SLPI* were higher in OVX than in intact females 3 days post‐CTX injury (Liao et al., 2019).

E2 supplementation in intact males (Tiidus & Bombardier, 1999) and E2 replacement in OVX females showed reduced MPO activity in lower limb muscles during the first 24 h post downhill running. In addition, E2 replacement in females reduced both neutrophil (visually identified by hematoxylin and eosin staining) (Tiidus et al., 2001) and leukocyte infiltration (i.e., His48^+^ or ED1^+^ cells) at 1 h–24 h post downhill running (Enns et al., 2008; Iqbal et al., 2008). On the contrary, leukocyte infiltration (i.e., 7/4^+^ or Ly6C/G^+^ cells) was increased in both myofibers and connective tissue after E2 replacement in response to repeated electrically‐evoked lengthening contractions (Fulkerson et al., 2015). In the same way, the expression of several genes encoding chemokines (*Ccl2*, *Ccl6*, *Ccl24*, *Ccr1*, *Cxcl1* and *Cxcl5*), known to modulate neutrophil and macrophage recruitment, was upregulated during the first day post freeze injury (Le et al., 2018). This was associated with a higher proportion of neutrophils (i.e., CD11b^+^LY6G^+^ cells) in injured muscles while the percentage of macrophages (i.e., CD11b^+^CD68^+^ cells) was not affected. Treated females also displayed a faster force recovery, even though the relation between the immunomodulatory effects of E2 and the functional improvement was not directly demonstrated (Le et al., 2018).

#### 3.1.3 Modulation of ER

A specific ER inhibition using ICI 182780 had no impact on the attenuation of leukocyte infiltration (i.e., His48^+^ or ED1^+^ cells) induced by E2 treatment in females 1 day after downhill running (Enns et al., 2008). Also, mean fluorescent intensity of CD11b^+^ and F4/80^+^ cells was higher in both OVX mice and females treated with a selective ER modulator (i.e., 4‐Hydroxytamoxifen) as compared with untreated animals 3–7 days after CTX injury (Liao et al., 2019). However, the biological effect of this increase on skeletal muscle regeneration remains unclear. Treatment with ER‐α and ER‐β agonists suppressed the elevation of both gene and protein expression of TNF‐α (i.e., a pro‐inflammatory cytokine) induced by OVX 1 day after NTX injury in females (Velders et al., 2012). In addition, mRNA expression of *Ccl2* and *Il10* was upregulated by ER‐β agonist 1–3 days post‐NTX while these markers were unaffected by ER‐α agonist (Velders et al., 2012). The same study also showed that mRNA expression of *Tnf* was not different between injured muscles of WT, ER‐α KO and ER‐β KO females at a later timepoint (i.e., 3 days post‐NTX) while *Ccl2* mRNA expression was lower in injured muscle of ER‐β KO mice as compared with ER‐α KO and WT animals.

To conclude, there is so far no consensus on the role of ovarian hormone deficiency and E2 replacement/supplementation/modulation on the inflammatory process during skeletal muscle regeneration.

### 3.2 Human studies

Only a few human studies investigated the influence of sex or E2 on the regulation of the inflammatory process (Figure 2).

#### 3.2.1 Effect of sex

The presence of radiolabeled Tc‐99 m neutrophils (quantified by invivo imaging) was greater in women as compared with men 2 h after repeated lengthening contractions (MacIntyre et al., 2000). On the contrary, and despite the same extent of muscle damage (i.e., quantified by z‐line streaming) and similar increase in CK activity, men showed a higher number of leucocytes in muscle tissue (i.e., cells positive for leucocyte common antigen) and circulating granulocytes as compared with women 2 days after lengthening contractions (Stupka et al., 2000). Also, the mRNA expression of *CXCL8* and *CD11* was similarly upregulated in men and women while a trend toward a greater increase of *CCL2* gene expression was found in men as compared with women 2 days after repeated lengthening contractions (Fortino et al., 2022). The number of macrophages (i.e., CD68^+^ cells) increased similarly while the number of leukocytes (i.e., cells positive for MPO) remains unchanged in men and women 1 day after a single bout of lengthening contractions leading to the same extent of muscle damage (i.e., quantified by z‐line streaming and torque recovery). However, when the same damaging exercise was repeated 5–6 weeks later, women displayed an increase of MPO^+^ cells while this parameter remains unchanged in men (Stupka et al., 2001), suggesting a faster inflammatory response in women as compared with men.

Overall, the influence of sex on the inflammatory process is still controversial in humans.

#### 3.2.2 Effect of E2 supplementation in men and postmenopausal women

E2 supplementation in men attenuated the infiltration of leukocytes (i.e., MPO^+^ cells) but did not protect against macrophage infiltration (i.e., CD68^+^ cells) 2 days after lengthening contractions (MacNeil et al., 2011). However, E2 supplementation also resulted in a reduction of circulating testosterone concentrations so that these two hormones could have contributed to the lower recruitment of leukocytes in the injured muscle (MacNeil et al., 2011). Interestingly, postmenopausal women using hormonal therapy displayed a lower increase in *IL6*, *IL8*, *IL15*, and *TNFA* mRNA expression and in CK activity as compared with women not using hormonal therapy after repeated lengthening contraction (Dieli‐Conwright et al., 2009). However, it is unclear how and to what extent hormonal therapy really improves muscle regeneration in humans through the modulation of the inflammatory response inasmuch as the investigations were limited to the first 4 h postexercise. Further studies are therefore needed to define the role and mechanisms of action of sex and E2 on muscle inflammation.

## 4 METHODOLOGICAL CONSIDERATIONS AND PERSPECTIVES

The substantial differences in the study design in both animal and human studies often make difficult the interpretation of the role of sexual dimorphism on skeletal muscle regeneration. The limitations of these different experimental conditions as well as the alternative approaches to most effectively evaluate the impact of sex/estrogen on skeletal muscle regeneration are summarized in Table 1.

**TABLE 1:** Limitations of the current experimental conditions and alternative approaches to most effectively evaluate the impact of sex/estrogen on skeletal muscle regeneration.

Experimental design	Limitations	Alternative approaches
Ovariectomy	Ovariectomy disturbs whole body homeostasis. Several hormones are altered following removal of the ovaries	Use of speficic ER knockout‐mice. This would also allow to keep intact the ovarian cycle of females
E2 replacement/supplementation	Use of supraphysiological E2 doses (i.e., ranging from 100 to 200 pg/mL)	Use of physiological E2 doses (i.e., 10–60 pg/mL). Consider the natural cyclic variations of E2 during the ovarian cycle (Byers et al., 2012; McLean et al., 2012)
ER agonists treatment	ER‐β agonist concentration was ten‐fold higher than the concentration of ER‐α agonist (Velders et al., 2012)	Use similar concentrations for treatment with ER‐β agonist and ER‐α agonist
Kinetics of skeletal muscle regeneration	Often limited to a single (and early) timepoint post‐injury	Consider multiple timepoints that cover the different key steps of myogenesis and inflammation
Cell culture	Assessment of SC fate in culture media with fetal bovine serum or chick embryo extracts, which contain growth factors and steroids such as E2 (Kitajima & Ono, 2016; Seko et al., 2020). Use of phenol red, as an indicator of bacterial contaminations, which is also known as a selective modulator of ER‐β due to its structure very close to that of non‐steroidal E2 (Berthois et al., 1986; Welshons et al., 1988)	Use phenol‐free medium and charcoal stripped serum to avoid interactions between medium components and estrogens (Larson et al., 2022)
Characterization of cell types/status	Lack of specificity of markers used to characterize leukocyte infiltration	Use of specific markers for immunostaining Omic approaches (single‐cell/single‐nucleus RNA seq, spatial transcriptomics) to identify a subpopulation of myogenic/inflammatory cells and/or specific transcriptomic signature
Model of muscle damage	Use of different models (i.e., downhill running, electrically‐evoked or voluntary lengthening contractions, CTX, NTX) leading to different magnitude of muscle injury (from minor to severe) Muscle damage was usually quantified with indirect markers such as blood parameters (i.e., CK activity), which do not reflect the extent of the damage	Control the magnitude of muscle injury using force measurements considered as the best indirect marker of muscle damage (Clarkson & Hubal, 2002; Fouré & Gondin, 2021) Use of a standardized model of muscle injury allowing the modulation of muscle damage severity (Bernard et al., 2023)

## 5 CONCLUSIONS

Growing evidence is emerging regarding a sexual dimorphism in SC regulation after muscle damage, illustrating a positive effect of E2 on muscle regeneration. Data are however still conflicting on the impact of sex and E2 on the inflammatory response. Additional studies are therefore warranted to improve our understanding of the influence of sex and E2 on the modulation of the inflammatory process. Animal investigations should allow to understand how E2 influence the interactions between SCs and immune cells in the regulation of muscle regeneration, a key process which has been so far mainly described in males (Arnold et al., 2007; Mounier et al., 2013). In addition, studies should carefully control the influence of ovarian cycle variation in female sex hormones, hormonal contraception as well as the natural drop of E2 in postmenopausal women on the regulation of both SC and immune cells after skeletal muscle injury. This would be of utmost interest for improving the management of muscle regeneration in healthy women and in pathological conditions associated with E2 deficiency/modulation (e.g., sarcopenia, hormone therapy for breast cancer…).

## AUTHOR CONTRIBUTIONS

CJ and JG contributed to the conception and design of this review. CJ and JG wrote the draft manuscript. All authors contributed to and revised the final manuscript. All authors read and approved the final manuscript.

## ETHICS STATEMENT

None.

## FUNDING INFORMATION

This work was supported by a PhD fellowship from GDR CNRS Sport et Activité Physique to CJ.

## Supporting information


**Data S1:** Supporting Information
