# Supplementary material for: Influence of sexual dimorphism on satellite cell regulation and inflammatory response during skeletal muscle regeneration
Source: Physiol Rep. 2023 Oct 5;11(19):e15798. doi: 10.14814/phy2.15798 (PMC10555529; doi:10.14814/phy2.15798)
Supplement: Supplementary file 1 — Data S1: Supporting Information [file PHY2-11-e15798-s001.docx]

Lyon, August 2^nd^, 2023

Dear Editor-in-Chief,

On behalf of my colleague, I am submitting the revised version of our review entitled: “Influence of sexual dimorphism on satellite cell regulation and inflammatory response during skeletal muscle regeneration” in which we address how sex and estrogens regulate both the satellite cell fate and the inflammatory response during skeletal muscle regeneration.

We would like to thank you for the positive evaluation of our work. We have addressed your minor comment. Overall, changes are highlighted in yellow in the revised manuscript.

We hope that our manuscript will be considered for publication in *Physiological Reports.*

Yours sincerely,

Julien Gondin, PhD
